# Supplementary material for: OpenMM 7: Rapid development of high performance algorithms for molecular dynamics
Source: PLoS Comput Biol. 2017 Jul 26;13(7):e1005659. doi: 10.1371/journal.pcbi.1005659 (PMC5549999; doi:10.1371/journal.pcbi.1005659)
Supplement: S1 Source Code — (ZIP) [file pcbi.1005659.s002.zip › OpenMM-7.0.1-Source/docs/api-python/generated/simtk.openmm.app.pdbreporter.PDBReporter.html]

PDBReporter — OpenMM 7.0.1.dev-5e86c4f documentation


# PDBReporter¶

*class* `simtk.openmm.app.pdbreporter.``PDBReporter`(*file*, *reportInterval*)¶
:   PDBReporter outputs a series of frames from a Simulation to a PDB file.

    To use it, create a PDBReporter, then add it to the Simulation’s list of reporters.

    `__init__`(*file*, *reportInterval*)¶
    :   Create a PDBReporter.

        |  |  |
        | --- | --- |
        | Parameters: | - **file** (*string*) – The file to write to - **reportInterval** (*int*) – The interval (in time steps) at which to write frames |

    Methods

    |  |  |
    | --- | --- |
    | `__init__`(file, reportInterval) | Create a PDBReporter. |
    | `describeNextReport`(simulation) | Get information about the next report this object will generate. |
    | `report`(simulation, state) | Generate a report. |

    `describeNextReport`(*simulation*)¶
    :   Get information about the next report this object will generate.

        |  |  |
        | --- | --- |
        | Parameters: | **simulation** (*Simulation*) – The Simulation to generate a report for |
        | Returns: | A five element tuple. The first element is the number of steps until the next report. The remaining elements specify whether that report will require positions, velocities, forces, and energies respectively. |
        | Return type: | tuple |

    `report`(*simulation*, *state*)¶
    :   Generate a report.

        |  |  |
        | --- | --- |
        | Parameters: | - **simulation** (*Simulation*) – The Simulation to generate a report for - **state** (*State*) – The current state of the simulation |

    `__delattr__`¶
    :   x.\_\_delattr\_\_(‘name’) <==> del x.name

    `__format__`()¶
    :   default object formatter

    `__getattribute__`¶
    :   x.\_\_getattribute\_\_(‘name’) <==> x.name

    `__hash__`¶

    `__reduce__`()¶
    :   helper for pickle

    `__reduce_ex__`()¶
    :   helper for pickle

    `__repr__`¶

    `__setattr__`¶
    :   x.\_\_setattr\_\_(‘name’, value) <==> x.name = value

    `__sizeof__`() → int¶
    :   size of object in memory, in bytes

    `__str__`¶

High performance molecular simulation on GPUs


### Navigation

- Application Layer
  - Loaders and Setup
  - Representation and Manipulation
  - Simulation
  - Reporting Output
  - Extras
- Library Layer

©2015, Stanford University and the Authors.
|
Powered by Sphinx 1.3.1
& Alabaster 0.7.7
|
Page source
